# Visual and Demographic Risk Factors for Falls and the Impact of Cataract Surgery in Elderly Patients

**DOI:** 10.18502/jovr.v19i3.13536

**Published:** 2024-09-16

**Authors:** Hamid Reza Heidarzadeh, Akbar Derakhshan, Seyed Hossein Ghavami Shahri, Mohammad Reza Ansari Astaneh, Elham Bakhtiari, Saeed Shokouhi Rad, Mojtaba Abrishami

**Affiliations:** ^1^Eye Research Center, Mashhad University of Medical Sciences, Mashhad, Iran; ^3^Hamid Reza Heidarzadeh: https://orcid.org/0000-0003-2811-9073

**Keywords:** Cataract Surgery, Fall, Older Adults, Risk Factors

## Abstract

**Purpose:**

To evaluate the effect of cataract surgery and visual impairment and the associated risk factors on the frequency of falls among older adults in northeast Iran.

**Methods:**

This cross-sectional study, conducted between 2019 and 2020, analyzed the potential risk factors of falling in older adults over 50 years of age. To this end, 380 patients were randomly selected by convenience sampling and classified into two groups: those who had undergone cataract surgery in the last 12 months (surgery group) and those who had not (cataract group). The data were collected from the medical records and face-to-face interviews, and logistic regression was used to identify potential risk factors for falling.

**Results:**

The frequency of falls in the cataract and surgery groups was 18.9% and 11.6%, respectively. The mean decimal visual acuity of the dominant eye was significantly lower in the cataract group than in the surgery group (*P* < 0.001). There were no significant differences in the mean number of medications used, Charlson Comorbidity Index score, Instrumental Activities of Daily Living score, and 10-Meter Walk Test speed between the two groups. According to the results of backward logistic regression, taking more than four medications per day and slow gait speed were the most important factors influencing the frequency of falls in older adults.

**Conclusion:**

Logistic regression analysis indicated that undergoing cataract surgery is not a significant protective factor against falls. However, older adults in the surgery group experienced fewer falls than in the other group. Besides, the results suggest that taking more than four medications daily and having a slow gait speed are significant fall risk factors.

##  INTRODUCTION 

Fall is an unexpected event that causes a person to come to rest on the ground, floor, or another lower level.^[1]^ It is the second leading cause of unintentional injury death,^[2]^ accounting for 695,771 deaths in 2017 around the world.^[3]^ The global age-standardized incidence and prevalence of falls were 2238 and 5186 per 100,000 in 2017, respectively.^[3]^ In 2015, the age-standardized death ratedue to fallswas 2.13 per 100,000 in Iran,^[4]^ and the incidence ofall fall-related injuries was 59 per 1000 person-year.^[5]^

The main domains of fall risk factors among older adults include (1) balance and mobility, (2) environmental, psychological, and medical conditions, (3) medication, and (4) sensory, neuromuscular**, **and sociodemographic factors.^[6]^ Other risk factors associated with falls include older age, female gender, being unmarried or widowed, lower education, worklessness, and low satisfaction with one's partner.^[7]^

In this context, previous studies have considered visual impairment (VI) as one of the critical risk factors for falls.^[8]^ Age-related cataracts are the leading cause of VI among older adults, and cataract surgery is the standard curative treatment option, offering a 90% success rate.^[9]^ Phacoemulsification is the most popular surgical method for cataract surgery. The number of cataract surgeries has tripled in the past four decades, mainly due to the aging population.^[10]^ In the Blue Mountain Eye Study, poor visual acuity (VA), reduced contrast sensitivity, and decreased visual field were reported as crucial visual risk factors for falls.^[11]^

Several studies have shown mixed results regarding the association between cataract surgery and falls. While some suggest that it can reduce the incidence of falls, others have found no significant correlation.^[8,10,12,13,14,15,16,17,18,19,20,21]^ This discrepancy could be due to factors such as differences in study methods, participants' age and race, selection biases, environmental factors, and other controllable and uncontrollable confounding factors. In the present study, we aimed to evaluate the effect of cataract surgery and VI and other associated risk factors on the frequency of falls among older adults.

##  METHODS

### Setting and Study Population

This cross-sectional study was performed on all patients with cataracts, aged over 50, who were referred to Khatam-al-Anbia Hospital between 2019 and 2020. Participants were selected consecutively using convenience sampling. The study protocol adhered to the tenets of the Declaration of Helsinki. The Regional Committee on Medical Ethics approved the ethical aspects of the study at Mashhad University of Medical Sciences, Mashhad, Iran (IR.MUMS.MEDICAL.REC.1399.761). Besides, a written informed consent was obtained from all participants. The information was collected through face-to-face interviews and reviewing patients' medical records. In this study, patients were visited only once. In some cases, due to a lack of information, it was necessary to communicate further with the patients, which was fulfilled using a telephone interview. The collected data included age, gender, number of comorbidities, number of medications used, daily physical activity, and walking speed.

The exclusion criteria included any history of other ocular diseases that could affect VA, such as clinically significant glaucoma, retinal detachment, amblyopia, proliferative diabetic retinopathy, diabetic macular edema, retinal degenerations and dystrophies, ocular abnormalities, and optic nerve disorders.

Participants meeting the eligibility criteria were divided into the surgery and non-surgery (cataract) groups. All patients in the cataract group had age-related cataracts with clinically significant decreased vision that was not corrected with spectacles. The surgery group consisted of individuals who had uncomplicated cataract surgery, either unilateral or bilateral, more than a year ago. All patients could walk independently without needing assistive devices such as canes, walkers, crutches, or braces.

In this study, cataract surgery was the exposure of interest, and the occurrence of falls was the primary outcome of interest.

### Assessment 

#### Visual acuity (VA)

The dominant eye best-corrected VA was recorded, in decimal, as the patient's VA. Then, we divided the patients into three VI groups. Patients with a VA ≥ 0.5 were classified as "No VI", from 0.5 to ≥ 0.3 as "Mild VI", and < 0.3 as "Moderate to severe VI".^[22]^

#### Fall

During their first visit, patients were asked about the occurrence of falls in the last 12 months. We described falls as “an unexpected event in which a person comes to rest on the ground, floor, or other lower level.”^[1]^ We excluded expected and unavoidable falls such as pushing, car accidents, and sliding. Patients with ≥ 2 falls were considered fallers. We also asked about the severity of falls and categorized them as “without injury,” “injuries that required outpatient treatment,” and “injuries that required inpatient care.”

#### Charlson Comorbidity Index (CCI) 

We used CCI to score patients' systemic disease profiles and measure the burden of comorbid diseases.^[23]^ CCI evaluates 19 chronic diseases and assigns a weight to each one. The sum of scores demonstrates the risk of mortality. We divided the patients into four groups with scores “0”, “1–2”, “3–4”, and “ ≥ 5.”

#### Instrumental Activities of Daily Living (IADL)

The IADL index was used to evaluate seven items on a patient's ability to live independently: using the phone, managing medicines, cooking, housework, transportation, doing the laundry, and managing finances. Each item has three dependent options: low function or dependent (zero points), needs help (1 point), and high function or independent (2 points). The sum of scores is from 0 to 14, and the score of "0–6" is classified as “Dependent”, “7–10” as “Need some help”, and “10–14” as “Independent.”^[24]^ The reliability and validity of the Persian version of IADL have been measured before.^[25]^ The content validity index, Cronbach's alpha, and ICC were reported to be more than 0.82 and 0.75, respectively.

#### 10-Meter Walk Test (10MWT)

We evaluated the patient's comfortable gait speed using the 10MWT scale. Accordingly, patients walk without assistance for 10 m, and the gait time is measured for the intermediate 6 m. The gait speed is then recorded in m/s.^[26]^

### Statistical Analysis

Data were analyzed using the SPSS version 16:0 (IBM SPSS Statistics, IBM Corporation, Chicago, Illinois). Quantitative variables were reported with mean and standard deviation, and qualitative variables with frequency and percentage. Independent-sample *t*-test or its non-parametric equivalent was utilized to compare quantitative variables, and the Chi-square test was used to compare qualitative variables between the two groups. Stepwise logistic regression (backward-conditional) was used to find the most important risk factors associated with falls. Due to the collinearity between VI and study groups, only study groups were entered into the model. The statistical significance level was set at 0.05.

**Table 1:** Demographic, medical, and functional characteristics of study participants.


**Characteristic**	**Cataract group (** ***n*** ** = 190)**	**Surgery group (** ***n*** ** = 190)**	***P*** **-value**
Age (yr), mean ± SD	67.83 ± 10.75	67.03 ± 9.77	0.92
50–64, *n* (%)	81 (42.6)	79 (41.6)	0.17
65–79, *n* (%)	70 (36.8)	84 (44.2)	
≥ 80, *n* (%)	39 (20.5)	27 (14.2)	
Female, *n* (%)	102 (53.7)	93 (48.9)	0.35
Dominant eye decimal VA, mean ± SD	0.24 ± 0.14	0.92 ± 0.11	< 0.001
Dominant eye LogMAR, mean	0.61	0.03	
VI groups, *n* (%):		
No VI	5 (2.6)	190 (100)	
Mild VI	90 (47.4)	0 (0)	< 0.001
Moderate to severe VI	95 (50)	0 (0)	
Faller, *n* (%)	36 (18.9)	22 (11.6)	0.04
Fall severity, *n* (%):		
No injury	21 (58.3)	15 (68.2)	
Inpatient	12 (33.3)	5 (22.7)	0.68
Outpatient	3 (8.33)	2 (9.1)	
Medications, mean ± SD	2.9 ± 2.41	3.24 ± 2.21	0.12
≤ 4, *n* (%)	131 (68.9)	120 (63.2)	0.23
> 4, *n* (%)	59 (31.1)	70 (36.8)	
CCI score, mean ± SD	1.87 ± 1.85	1.94 ± 1.65	0.67
0, *n* (%)	46 (24.2)	40 (21.1)	0.81
1–2, *n* (%)	94 (49.5)	95 (50)	
3–4, *n* (%)	33 (17.4)	39 (20.5)	
≥ 5, *n* (%)	17 (8.9)	16 (8.4)	
IADL score, mean ± SD	11.29 ± 2.5	11.12 ± 2.62	0.9
Dependent, *n* (%)	11 (5.8)	15 (7.9)	0.71
Need some help, *n* (%)	49 (25.8)	49 (25.8)	
Independent, *n* (%)	130 (68.4)	126 (66.3)	
10-MWT (m/s), mean ± SD	0.97 ± 0.14	0.98 ± 0.16	0.33
	
	
VA, visual acuity; VI, visual impairment; CCI, Charlson Comorbidity Index; IADL, Instrumental Activities of Daily Living; 10-MWT, 10-Meter Walk Test			

**Table 2:** Summary of the backward stepwise (conditional) logistic regression analysis (first and final steps) for the risk factors of falls.


	**Step 1**			** Step 5**		
**Variable**	**Odds ratio of falling**	**95% CI**	***P*** **-value**	**Odds ratio of falling**	**95% CI**	***P*** **-value**
Age ( > 65)	0.998	0.969–1.027	0.872		
Gender (Female)	0.942	0.514–1.725	0.847		
History of cataract surgery (Positive)	0.482	0.261–0.893	0.020	0.490	0.267–0.897	0.210
Medications use ( > 4)	1.189	1.024–1.380	0.023	1.189	1.043–1.355	0.009
CCI score	1.042	0.828–1.312	0.726		
IADL score (independent)	0.658	0.260–1.667	0.378		
10-MWT	0.010	0.001–0.170	0.001	0.120	0.001–0.109	< 0.001
	
	
CCI, Charlson Comorbidity Index; IADL, Instrumental Activities of Daily Living; 10-MWT, 10 Meter Walk Test; yr, year						

##  RESULTS

In total, 380 patients with a mean age of 67.43 ± 10.27 (range, 50–89 years) were enrolled in the study. Of these, 195 cases (51.3%) were female and 185 (48.7%) were male. There were two groups (*n* = 190 each) of cataract and surgery. The mean age of the cataract and surgery groups was 67.83 ± 10.75 and 67.03 ± 9.77 years, respectively. The number of females in the cataract and surgery groups was 102 (53.7%) and 93 (48.9%), respectively. Hence, there was no statistically significant difference between the two groups regarding age and gender (*P *= 0.92 and *P *= 0.35, respectively).

The mean VA of the dominant eye in the cataract and surgery groups was 0.24 ± 0.14 and 0.92 ± 0.11, respectively, showing a statistically significant difference (*P* < 0.001). No patient in the surgery group (*n* =190) had a VI, while in the cataract group, 5 (2.6%) had no VI, 90 (47.4%) had mild VI, and 95 (50%) had moderate to severe VI. The difference was also statistically significant between the two groups (*P* < 0.001).

Thirty-six (18.9%) patients in the cataract group reported falling, compared with 22 (11.6%) patients in the surgery group. While the two groups showed a statistically significant difference in the number of falls (*P *= 0.04), they did not differ significantly in terms of fall severity (*P *= 0.68).

Fifty-nine (31.1%) patients in the cataract group were taking more than four medications per day, compared with seventy (36.8%) patients in the surgery group (*P *= 0.23). Regarding the CCI score, IADL score, and 10-MWT, both groups were approximately the same (*P *= 0.81, 0.71, 0.33, respectively) [Table 1].

Table 2 summarizes the results of the backward stepwise (conditional) logistic regression analysis (first and final steps) for the risk factors of falls. It can be observed that gender, age, history of cataract surgery, medication use, CCI score, IADL score, and 10-MWT were included in the model at the first step. However, age, gender, CCI score, and IADL score were excluded from the model in the second, third, and fourth steps, respectively. The final step was devoted to the history of cataract surgery, medication use, and 10-MWT. In this last step, the only variables with statistical significance were medication use (odds ratio [95% CI] = 1.189 [1.043–1.355], *P *= 0.009) and 10-MWT (odds ratio [95% CI] = 0.120 [0.001–0.109], *P* < 0.001) [Table 2].

##  DISCUSSION

Falls are the second leading cause of unintentional injury death,^[2]^ and there is a need for strategies to prevent falls among older adults. Although many previous studies have examined the effect of cataract surgery on the incidence of falls, the results are controversial.

The present study showed that cataract surgery is not associated with the frequency of falls in older adults. We also found that using more than four medications daily and slow gait speed are the risk factors for falls in old patients. On the other hand, we found no significant relationship between the risk of falls and the patient's gender and age.

Our results confirm those studies that have reported no association between cataract surgery and falls.^[19,20,21]^ A retrospective cohort study on 13,385 patients showed that pseudophakic monovision has no impact on fall risk, but aiming for emmetropia increases falls compared to cataract patients. They also found older age, female sex, and preexisting myopia as fall risk factors.^[19]^ Another study on 214 patients demonstrated that cataract surgery did not affect fall risk and patient mobility.^[20]^

In contrast, there are reports on the positive impact of cataract surgery in reducing falls among older adults.^[8,10,13,14,15,16,17,18]^ A prospective clinical study by Brannan et al on 84 patients indicated that cataract surgery reduced the risk of falls postoperatively compared with preoperatively. They also reported that a history of falls, using assistive devices, and taking more than four medications are risk factors for falls in older adults with VI due to cataracts.^[8]^ In our study, we excluded patients who were using assistive devices. However, taking more than four medications was a fall risk factor in our study as well.

A prospective longitudinal cohort study on 55 older adults reported a significant decrease in the risk of falls after the first (54%) and second cataract surgeries (73%) compared with the period before the first cataract surgery. Living alone was found to be a fall risk factor, and binocular low VA was strongly associated with fall risk. They also evaluated the impact of sufficient exercise and the number of medications on fall risk, but they did not find a significant association in this respect.^[10]^

In another cohort study on 409 individuals aged 65 years old or more, Keay et al evaluated the incidence of falls after the first and second cataract surgeries. They found that although cataract surgery improved vision in the first eye, it was necessary to perform cataract surgery in the second eye as well in order to reduce the incidence of falls.^[12]^

We did not categorize patients based on whether they had cataract surgery done in one or both eyes. This is a limitation of our study since being pseudophakic in one eye may result in anisometropia and imbalance. However, a recent study confirmed the first cataract surgery significantly improved patients' balance.^[27]^ Another limitation of our study was that we did not employ random sampling, and it could lead to sampling bias. Additionally, a more extensive study involving more patients could result in more conclusive evidence regarding the true relationship between falls and their risk factors. Finally, our study did not account for several other factors, like postoperative refraction, postoperative complications, intraocular lens material, patient occupation, level of education, marriage status, place of residence, and others, that may have influenced the incidence of falls among the study population.

To summarize, our study utilized stepwise logistic regression to identify the impact of factors such as age, gender, medication use, CCI score, IADL score, and gait speed, in addition to cataract surgery, on the frequency of falls among older adults. The results suggested that cataract surgery was not associated with the frequency of falls. On the other hand, taking more than four medications daily and slow gait speed were among the most potential risk factors for falls in old patients.

### Financial Support and Sponsorship

The authors would like to acknowledge the financial support of the Vice-Chancellor of Research of Mashhad University of Medical Sciences for this research project (code: 991473). The funding organization had no role in the design or conduct of this research.

### Conflicts of Interest

None.
